# Reflections on the EMBO Workshop: Neuroscience of Sleep 2025

**DOI:** 10.1242/bio.062079

**Published:** 2025-07-28

**Authors:** Rahul Kumar, Ritika Mukherji, Kamakshi Singh

**Affiliations:** ^1^ Department of Biology, Ashoka University, Plot no.2, Rajiv Gandhi Education City, Sonipat, Haryana 131029, India; ^2^ University of Oxford, Sherrington Building, Sherrington Rd, Oxford OX1 3PT, UK; ^3^ Tata Institute of Fundamental Research, Dr Homi Bhabha Rd, TIFR, Navy Nagar, Colaba, Mumbai, Maharashtra 400005, India

**Keywords:** Sleep, Neuroscience, Circadian, Behaviour, Memory, Ecology

## Abstract

The inaugural EMBO workshop on the neuroscience of sleep took place from 11th−13th March 2025 at the India Habitat Centre in New Delhi, India, and marked a milestone for the global sleep research community. It overlapped with the 25th anniversary of the discovery of sleep in *Drosophila*, and the meeting celebrated the scientific advances in recognising sleep as a deeply conserved and biologically vital process. With around 85 participants from across the globe, the workshop brought together scientists to explore the biological, molecular, and computational dimensions of sleep across scales. The conference program included keynote lectures from the pioneers in sleep research, exciting new studies on sleep's role in regulating neural computation, metabolism, and plasticity, and reflected the increasing interest in the field for sleep studies in non-model organisms in natural settings. In addition to the science, the conference involved thoughtful talks and conversations around women in science, gender equity, and varied career paths, with many participants sharing their own experiences. The meeting being hosted in India allowed space for meaningful exchange, collaboration, and mentorship among researchers of the Global South. This Meeting Review captures an overview of the scientific discussions that made this event a success.

## Teaser

**Summary:** The inaugural EMBO workshop on the neuroscience of sleep brought together a global sleep research community to discuss recent advances across species, scales and systems. The meeting explored different dimensions of sleep research and fostered exchange of interdisciplinary ideas, inclusivity and sustainability.

## Introduction

The conference showcased an extensive and well-curated program that covered a wide array of topics central to the field of sleep research. The sessions covered both fundamental and applied aspects of sleep science, ranging from molecular mechanisms to ecological and clinical perspectives. Talks on signalling pathways in the brains of model organisms like fruit flies, worms, and rodents provided valuable insights into the genetic and neurochemical regulation of sleep. Molecular genetics studies in classic model systems were complemented by studies of naturalistic sleep behaviours in wild species. Other talks discussed the influence of sleep and circadian dysregulation on human disease, metabolic and cardiac disorders. These talks, involving multi-level approaches, demonstrated how insights from basic research can help advance medical understanding and therapeutic strategies. A few talks also focused on the growing role of computational neuroscience in sleep research, particularly *in silico* modelling to understand sleep functions. Beyond the talks, the conference offered numerous interactive elements. Attendees had opportunities to engage in informal discussions with speakers during themed lunch tables, and the vibrant poster sessions enabled enthusiastic dialogue among early career and senior researchers. The EMBO Women in Science lecture addressed systemic biases in scientific institutions and sparked meaningful reflection on equity and inclusion in academia.

**Fig. 1.**
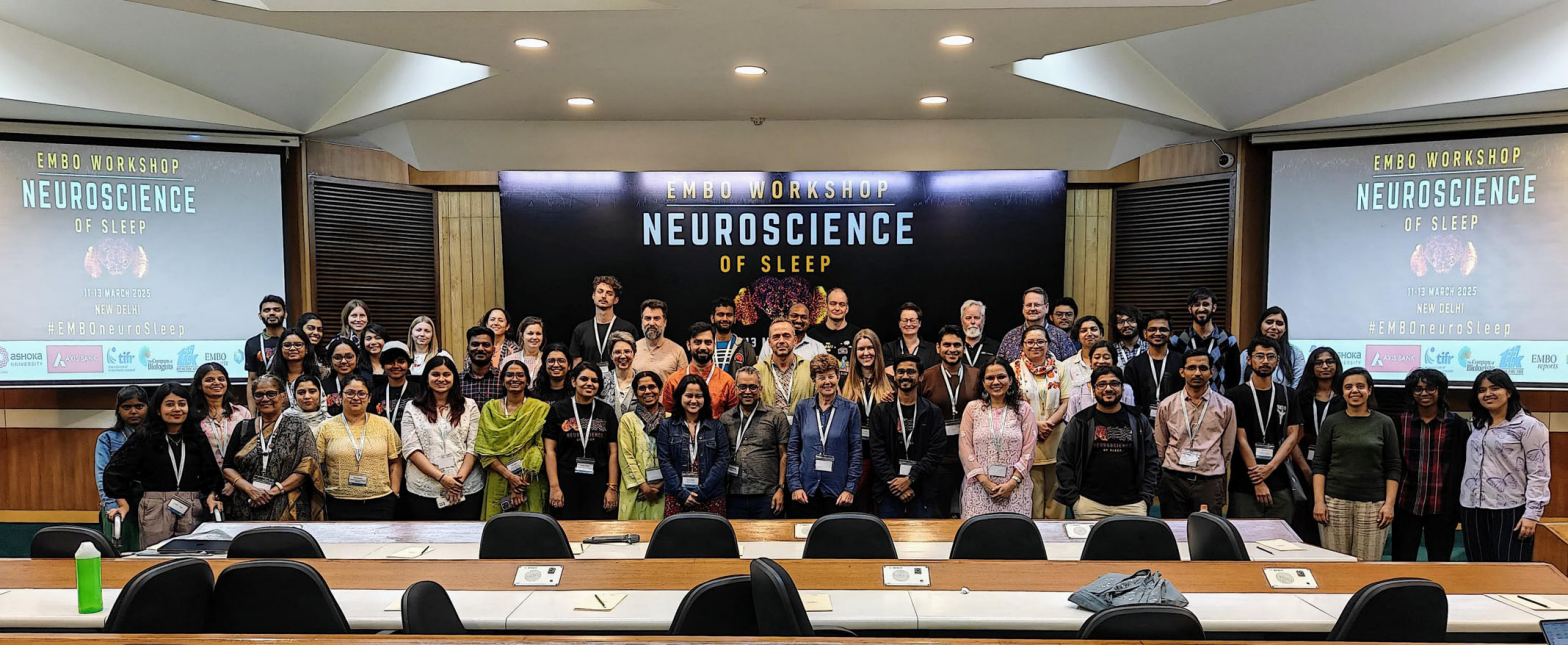
A group photograph of the in-person attendees for the EMBO Neuroscience of Sleep Workshop 2025, held at the India Habitat Centre, New Delhi, India.

## Discussing sleep across species and scales: from cells to circuits to behaviour

Sleep, in all its evolutionary complexity, was at the centre stage across different sessions of the conference. Perhaps no other physiological process exhibits such remarkable diversity across the animal kingdom (Pennisi, 2021), and many speakers emphasised the importance of investigating sleep across a wide range of model systems. From Arctic reindeer and octopuses to *C. elegans* and rodents, the meeting brought together both a diversity of species and the global community working to understand them.

Several talks highlighted how technological advances and better tools have enabled researchers to study sleep in field conditions, which more closely reflect real-world settings with enhanced precision and ease. These field-based studies are beginning to reveal that sleep is far more flexible and context-dependent than laboratory models suggest. For instance, one speaker presented work on tundra reindeer, showing that these animals may compensate for sleep loss through rumination and tend to reduce sleep during the months with lower light exposure. Other talks featured dramatic examples of sleep reduction, such as sandpipers during their mating season, unihemispheric sleep in migrating frigate birds and otariid seals, and torpor in Djungarian hamsters, emphasizing how ecological pressures shape sleep architecture. Studies on wild boars and fallow deer fawns, using high-resolution accelerometry, demonstrated substantial individual variability within species, and plastic responses of sleep over the annual cycle in relation to environmental conditions. Together, these findings point to the value of field studies in bridging the gap between controlled experiments and natural behaviours – a theme that resonated strongly throughout the meeting.

Marine and aquatic species featured prominently in discussions of sleep diversity. Talks comparing marine mammals and cephalopods revealed striking evolutionary contrasts in sleep expression, despite shared ecosystems. In octopuses and cuttlefish, speakers described active sleep states marked by dynamic skin patterning, elevated arousal thresholds, and homeostatic regulation – features that invited comparisons to REM sleep. Unihemispheric sleep in sea lions was shown to involve distinct patterns of eye closure and limb movement, prompting renewed interest in its underlying mechanisms. These presentations underscored the need to examine sleep with greater nuance in species whose behaviours challenge canonical sleep definitions.

**Fig. 2.**
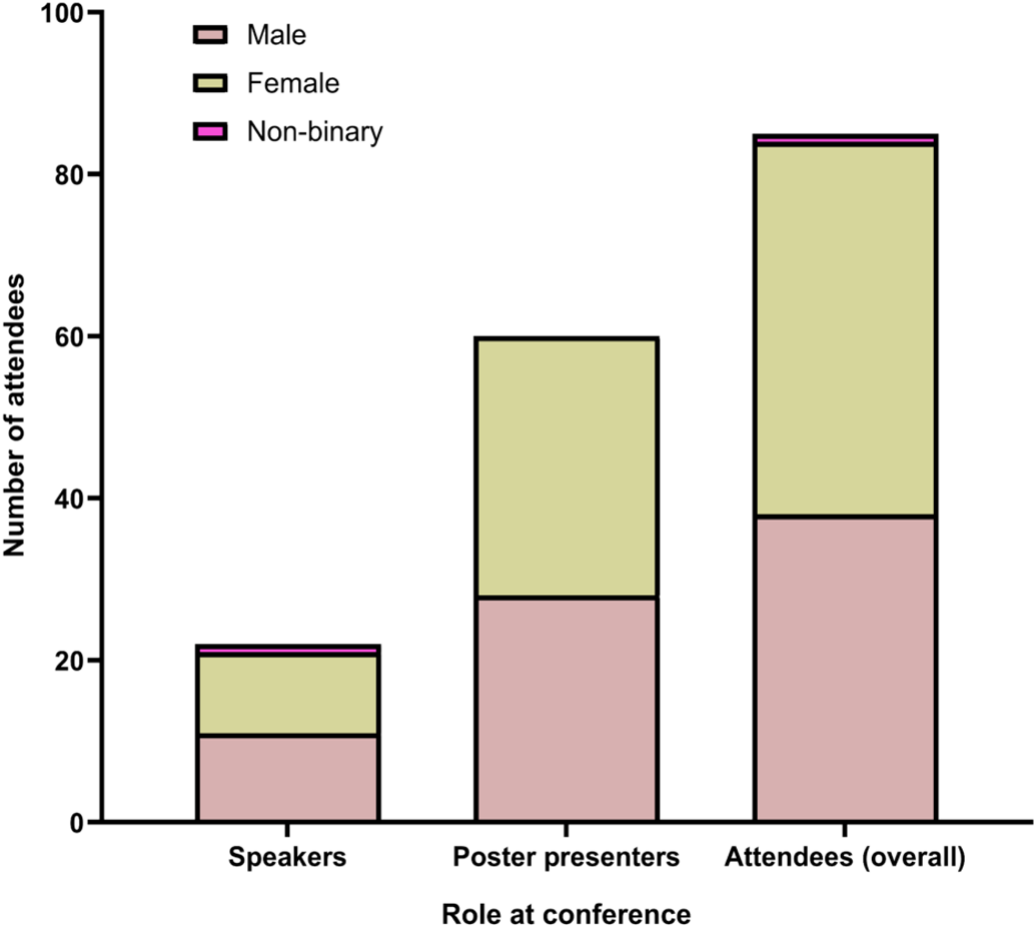
Gender distribution by attendee role at the EMBO Neuroscience of Sleep Workshop 2025.

We had the privilege of celebrating 25 years of sleep research in *Drosophila* with the founders of the field, Paul Shaw and Amita Sehgal. Both delivered remarkable and intriguing keynote lectures that highlighted the continuing contributions of fly studies to sleep science – a theme that other talks also reinforced. Amita spoke of a metabolic need for sleep required for orchestrating cellular maintenance in the fly brain and to prepare for subsequent wake. Paul presented work suggesting that circadian modulation restricts brain plasticity, and that enhancing sleep restored plasticity by overcoming this clock-imposed restriction. Glia might be one site where circadian modulation, sleep homeostasis and memory interact. Knockdown of certain clock genes in glia impaired homeostatic recovery sleep. Conversely, knockdown of certain antimicrobial peptides in glia resulted in an exaggerated sleep rebound and protection from sleep-loss-induced cognitive impairments. Other speakers also highlighted the emerging roles of astroglia in regulating sleep homeostasis in rodents.

Complementing studies on sleep regulation, several speakers explored emerging functions of sleep, including memory consolidation, synaptic homeostasis, development and immunity, sparking lively and thought-provoking discussions throughout the conference. The synaptic homeostasis hypothesis and the role of sleep in synapse maintenance were addressed in zebrafish larvae by repeated imaging of sparsely labelled single tectal cells. While synaptic downscaling was most evident during sleep, it was a subtype-specific phenomenon. This suggests that the role of sleep in synaptic maintenance is much more nuanced than a general downregulation. Parallel to these, selective synaptic remodelling was shown to occur in *C. elegans* undergoing memory consolidation. Sleep's role in memory consolidation was shown to be plastic: flies could shift between sleep-dependent and sleep-independent methods of memory consolidation based on nutrient availability. This flexibility suggests that sleep is adaptive to environmental conditions to ensure cognitive functionality. One compelling talk demonstrated that sleep helps maintain a universal principle of neural computation: criticality. At this critical state, neural populations exhibit maximal dynamic range, efficient information transmission, and computational adaptability. Criticality functions as a homeostatic set-point in the intact brain, with deviations from this state during wakefulness predicting subsequent sleep–wake behaviour. While extended wakefulness disrupts this computational optimum, sleep acts to restore it. Although most studies employ some form of sleep deprivation to address its relevance, thought is seldom given to the impact of the protocol itself. The deliberations at the workshop highlighted the evidence suggesting that specifics of the methods employed for sleep deprivation are non-trivial contributors to the results obtained. These diverse in-depth discussions about the functions and regulation of sleep, raised thought-provoking questions on how to better describe and define sleep and related states of vigilance in the future.

**Fig. 3.**
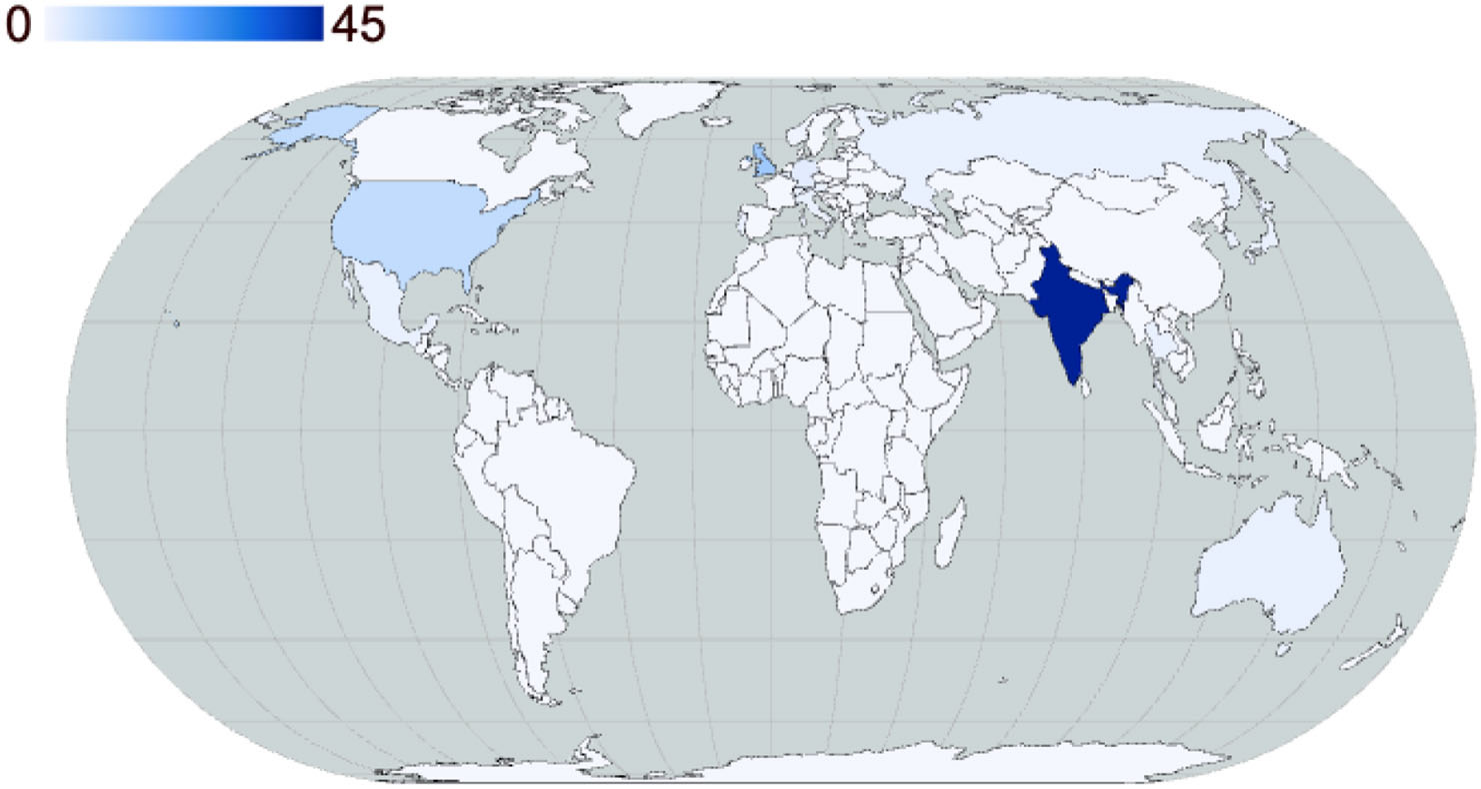
**Attendee diversity by citizenship at the EMBO Neuroscience of Sleep Workshop 2025.** (Made with https://app.flourish.studio/).

The workshop also discussed the consequences of our modern lifestyle on sleep. Experimental work mimicking night-shift conditions suggests drastic impacts on circadian rhythms, which potentially contribute to metabolic disruptions and compromised health. Clinical studies showed genetic overlap between sleep regulation and cardiovascular risk, using both patient-derived cells and model organisms. Others explored how sound therapy using soft, rhythmic sound waves known as ‘pink noise’ might improve sleep quality in aging populations, pointing towards simple, non-invasive approaches to better sleep and cognitive resilience. The time spent in N3 NREM sleep stage was shown to correlate with reduced anxiety-associated traits. The impact of these clinical and human studies can be immense if these can be translated into implementable changes. Fundamental research and clinical research can supplement each other and lead to great progress for the field.

Taken together, the workshop was successful in creating a multilayered picture of sleep. It emphasised that sleep is not just a passive state or a nightly shutdown, but an active, essential brain state, and intricately connected to almost every aspect of health and function.

## Beyond the science: people, planet, and purpose

The conference was hosted in New Delhi, India, with participants hailing from around the globe. Participation spanned faculty members, postdocs, PhD students, masters, and undergraduates. The participation of academics and students from the rest of the globe offered unique networking opportunities, particularly for early career researchers from the Global South. We hope that this conference sets a precedent for many such conferences to come. Nearly half of the invited speakers were women, symbolising that representation is improving and that science is stronger because of it. Though the future career trajectory is uncertain for many early career participants and students, mentorship and guidance were provided through a ‘career counselling session’ where early career researchers were encouraged to converse with more experienced scientists, not just about experiments, but about navigating the ups and downs of academic life, different career opportunities around the world and how to achieve a healthy work–personal life balance.

Sustainability was an important theme and was thoughtfully included in the event from the ground up. Talks were available in a hybrid format, making it easier for people to attend without long flights. Abstract books were digital, and there were no disposable water bottles or single-use cutlery included during the 3-day event. Train travel was encouraged over air travel wherever possible. These small but intentional choices reflected a growing awareness regarding how we gather as a scientific community matters just as much as what we discuss. The digital platform for the conference helped keep the discussion going even after the sessions ended, letting attendees ask follow-up questions and share ideas across time zones. In a world where connection often ends with the last slide, it was delightful to see dialogue continue long after the presentations. This thoughtful attention to people, planet and purpose made the workshop more than just a meeting. It felt like a model for how science can and should be done and discussed with the wider community.

## Conclusion and future directions

The meeting took place in a landmark year, marking 25 years of the foundational discovery of sleep in *Drosophila* (Shaw et al., 2000, and Hendricks et al., 2000). This milestone continues to shape the field by reinforcing the idea that sleep is a conserved and biologically significant phenomenon across species. One of the meeting's greatest strengths was its ability to bring together researchers from a wide range of disciplines and perspectives, fostering interdisciplinary dialogue and collaboration. The fact that such an event was hosted in India was particularly impactful, offering researchers from the Global South direct access and participation in the latest developments in sleep research. The meeting received highly positive feedback from attendees, and there is strong momentum to continue this initiative as a biennial event, helping to sustain and grow this vibrant fundamental sleep research community. Despite lasting a mere 3 days, the meeting has given shape to many collaborations and ideas that will far outlive the duration of the meeting, leaving us all with a renewed sense of curiosity and a hopeful glimpse into the exciting science that lies ahead.
